# Burden of antimicrobial resistance and estimated economic impact of *Klebsiella pneumoniae* in Iran— A 2000 to 2021 analysis

**DOI:** 10.1016/j.nmni.2025.101675

**Published:** 2025-11-20

**Authors:** Amirhossein Shahsavand, Ali Golestani, Samaneh Akbarpour, Mohammadreza Salehi, Arash Seifi, Keyhan Mohammadi, Maryam Shafaati

**Affiliations:** a Non-Communicable Diseases Research Center, Endocrinology and Metabolism Population Sciences Institute, Tehran University of Medical Sciences, Tehran, Iran; b Endocrinology and Metabolism Research Center, Endocrinology and Metabolism Clinical Sciences Institute, Tehran University of Medical Sciences, Tehran, Iran; c Sleep Breathing Disorders Research Center, Tehran University of Medical Sciences, Tehran, Iran; d Research Center for Antibiotic Stewardship and Antimicrobial Resistance, Infectious Diseases Department, Imam Khomeini Hospital Complex, Tehran University of Medical Sciences, Tehran, Iran; e Center for Communicable Disease Control, Ministry of Health and Medical Education, Tehran, Iran; f Department of Clinical Pharmacy, School of Pharmacy, Tehran University of Medical Sciences, Tehran, Iran

**Keywords:** Antimicrobial resistance, *Klebsiella pneumoniae*, Global burden of disease, Iran, Carbapenem resistance, Economic burden

## Abstract

**Background:**

Antimicrobial resistance (AMR) in *Klebsiella pneumoniae* (*K. pneumoniae*) poses a growing public health threat in Iran. This study assessed the burden of *K. pneumoniae* AMR from 2000 to 2021, including deaths, disability-adjusted life years (DALYs), and economic impact.

**Methods:**

Using 2021 AMR project data, we estimated deaths and DALYs per 100,000 population with 95 % uncertainty intervals under two scenarios: attributable (resistant replaced by susceptible infections) and associated (resistant replaced by no infection). Economic burden was calculated using GDP per capita and PPP-adjusted estimates.

**Results:**

The deaths from *K*. *pneumoniae* AMR increased from 565.9 to 643.5, while the age-standardized rate of deaths decreased from 1.5 to 0.9 per 100,000 people. DALYs dropped from 29523.4 to 18787.1, with the ASR decreasing from 60.2 to 25.3 per 100,000 people. Carbapenem resistance rose from 7.5 % to 20 %, increasing all-age attributable deaths from 76.1 to 175.0. Deaths and death rates fell for under-20s. For those 30 and over, death counts rose while the rate itself fell, stabilizing only for ages 85+. Despite a slight decrease, the economic burden was estimated at between 291.1 and 873.31 million USD PPP in 2021.

**Conclusions:**

While the overall burden of *K. pneumoniae* AMR has declined, it remains high in older adults. Strengthening **water, sanitation, and hygiene** (WASH) programs, antibiotic stewardship (ASP), and prescribing practices are essential.

## Highlights

• The burden of *Klebsiella pneumoniae* AMR in Iran increased, especially in older adults.
• Significant decline in *K. pneumoniae* AMR burden among children under five years.
• Carbapenem resistance rose from 7.5 % in 2000 to 20 % in 2021, increasing deaths.
• The economic burden of *K. pneumoniae* AMR in Iran was 291.1 million USD in 2021.

## 1 Introduction

Bacterial antimicrobial resistance (AMR) arises when bacteria undergo changes that reduce the effectiveness of antimicrobial agents used to treat infections [1]. Among antimicrobial-resistant bacteria, the sharp rise in infections caused by multidrug-resistant (MDR) and extremely drug-resistant pathogens from the *Enterobacteriaceae* family is a significant public health concern, as these bacteria are natural inhabitants of the human microbiome, and their infections are often linked to high mortality rates and heightened healthcare costs [2]. *Klebsiella pneumoniae*, a member of *Enterobacteriaceae*, is specifically well-known for its ability to develop AMR. Resistant *K*. *pneumoniae* contributes to approximately one-third of all Gram-negative infectious diseases, including bloodstream infections (BSIs), pneumonia, and urinary tract infections (UTIs), in both hospital-acquired and community-acquired settings [3]. Moreover, *K*. *pneumoniae* acts as a shuttle for spreading AMR genes from non-pathogenic to important, pathogenic bacteria, increasing the prevalence of AMR in other species, and contributing to the global AMR challenge [4].

Experts estimated in 2014 that AMR could lead to 10 million deaths by 2050. In response, the World Health Organization (WHO) issued a global action plan in 2015, aiming to tackle AMR worldwide, and the United Nations also included a decrease of AMR-associated blood infections as a sustainable development goal (3.d.2 indicator). Previous global burden of disease (GBD) studies in 2019 and 2021 [5,6] showed decreasing trends in the overall burden of AMR during the last 30 years; however, projections show that the burden of AMR will rise in the future, affecting people aged 70 years and older more severely. Globally, AMR of *K*. *pneumoniae* is the 2nd and 4th leading cause of all deaths associated and attributable to AMR, respectively. While a recent meta-analysis reported the prevalence of *K. pneumoniae* resistance in Iran [7] and several studies [[8], [9], [10]] have highlighted multidrug-resistant isolates, these efforts have not been translated to a national burden estimate. Local drivers, such as national antibiotic stewardship policies (ASPs), infection control capacity, and the prevalence of comorbidities, create distinct AMR trends that limit the direct application of global estimates for policymaking at the national level. Therefore, an evidence gap exists regarding the mortality, morbidity, and economic impact of *K. pneumoniae* AMR in Iran, which is essential for evidence-based health system planning.

To address this gap, our study provides the first comprehensive and comparable assessment of the burden of *K. pneumoniae* AMR in Iran from 2000 to 2021. Using the standardized methodology and extensive data sources of the GBD project, we estimated all ages and age-standardized deaths and DALYs, as well as the economic burden related to *K. pneumoniae* AMR and its related infectious syndromes. The burden of *K*. *pneumoniae* AMR was estimated based on two counterfactual scenarios, where resistant infection pathogens are replaced by susceptible pathogens or no infection at all, providing a useful insight into the effect of *K*. *pneumoniae* AMR burden in Iran.

## 2 Methods

### 2.1 Overview

This study utilized data from the 2021 AMR project developed by the Institute for Health Metrics and Evaluation (IHME). The AMR estimates were generated by integrating mortality and incidence data from the GBD 2021 study with a wide range of data sources, in order to quantify the burden of AMR associated with or attributable to 11 infectious syndromes, 22 bacterial pathogens, 16 antimicrobial drug classes, and 84 pathogen–drug combinations across 204 countries and territories. A comprehensive description of the methodology used in the AMR project is available elsewhere [5,6]. For the present analysis, we extracted data on *K*. *pneumoniae*-related AMR in Iran from the MICROBE (Measuring Infectious Causes and Resistance Outcomes for Burden Estimation) data platform, accessible at https://vizhub.healthdata.org/microbe/. All analyses were conducted in accordance with the Guidelines for Accurate and Transparent Health Estimates Reporting (GATHER) [11]. In this study, the authors independently conducted additional analyses, including the estimation of economic burden, calculation of percent change, and computation of estimated annual percent change (EAPC). All other results represent visualization and tabulation of publicly available data extracted from the IHME MICROBE platform.

### 2.2 Data sources

The AMR project incorporated a diverse range of data sources to estimate the burden of antimicrobial resistance. These included multiple cause-of-death records, hospital discharge data, outpatient and inpatient insurance claims, microbiological and laboratory data (both with and without linked outcomes), published literature, single-drug resistance profiles, antibiotic use data among children under five with reported illness, and mortality surveillance systems. Additionally, cause-of-death input data from the GBD study were used for modeling framework. In total, over 520 million individual data records spanning 19,513 location-years were utilized. A comprehensive description of the data sources and their inclusion process is available elsewhere [5].

### 2.3 Data processing and modelling

The AMR study followed a ten-step modeling framework to estimate the burden of antimicrobial resistance, which is briefly outlined here [5]. In the first two steps, the mortality envelope was established by identifying sepsis-related deaths using GBD 2021 cause-of-death estimates and applying mixed-effects logistic regression models to allocate deaths to 22 infectious syndromes—11 of which had sufficient data to estimate pathogen-specific AMR burden. Steps 3 and 4 focused on estimating pathogen-specific case fatality rates (CFRs) using the RegMod modeling environment and determining the distribution of pathogens across each infectious syndrome by incorporating data from over 24 million isolates. A multinomial modeling framework was employed to account for heterogeneity in data sources and covariates, including the Healthcare Access and Quality (HAQ) Index.

In steps 5 to 7, the prevalence of resistance for 84 clinically relevant pathogen–drug combinations were modeled using a two-stage spatiotemporal approach. This included adjustments for inconsistencies in laboratory interpretations and biases associated with facility types. National-level antibiotic consumption data were also modeled and used as covariates to improve estimation accuracy. To assess multidrug resistance, a novel optimization technique was applied to estimate the joint distribution of resistance patterns. Steps 8 and 9 estimated the relative risk of mortality and excess hospital stay for drug-resistant infections compared to susceptible ones using a meta-regression framework to obtain a population attributable fraction (PAF). Finally, in step 10, the AMR burden was estimated under two counterfactual scenarios: (A) attributable burden—assuming resistant infections were replaced by susceptible infections; and (B) associated burden—assuming resistant infections were replaced by no infection. Details of the methodology are provided in the ***Supplementary File V2.***

### 2.4 Health burden measures

In this study, we reported deaths and DALYs numbers and rates per 100,000 associated and attributable to *K. pneumoniae* AMR. Death attributable to resistance were calculated by multiplying several components: the number of cause-specific deaths, the proportion involving sepsis, the share of sepsis deaths linked to specific infectious syndromes, the proportion of those syndromes caused by particular pathogens, and the mortality PAF for each resistance profile. YLLs were then derived using GBD standard methods, which apply a counterfactual life expectancy based on age [12].

YLDs were calculated by combining the incidence of each infectious syndrome with the fraction caused by specific pathogens, the YLDs per case, and the non-fatal PAF. For resistance involving multiple antibiotic classes, the burden was redistributed across classes based on excess risk to ensure that each pathogen–drug combination had a mutually exclusive burden. DALYs were obtained by summing YLLs and YLDs. The total AMR burden in the drug-sensitive counterfactual scenario was calculated by aggregating burden across all pathogen–drug combinations. A similar approach was used to assess the associated burden of AMR, but instead of using mortality PAFs, the prevalence of resistance in deaths was applied. Uncertainty was propagated by incorporating 100 posterior distribution draws for each combination of location, sex, age group, and year at every analytical stage. These draws were used to generate the final estimates of deaths and infections both associated with and attributable to antimicrobial resistance. Uncertainty intervals (95 % UIs) were derived by applying a standard deviation of ±1.96 around the mean estimate [5]. The out-of-sample validity estimates were provided elsewhere [5].

### 2.5 Statistical analysis

To estimate the economic burden resulting from premature mortality and morbidity due to *K. pneumoniae*, we assigned a monetary value to DALYs. The Copenhagen Consensus has previously adopted gross domestic product (GDP) per capita as a standard proxy for approximating the monetary value of a DALY [13]. Commonly, one or three times the GDP per capita are used as reference values to estimate the economic value of a single DALY [14]. In line with established methodologies, we adopted one and three times the GDP per capita as approximate valuations of a single DALY [14]. For the base-case analysis, we used Iran's GDP per capita in 2021 (in USD). To facilitate international comparability, we also calculated estimates using GDP per capita based on purchasing power parity (PPP) for the same year. GDP data were obtained from the World Bank's World Development Indicators [15] and adjusted to per capita values using population estimates from the Global Burden of Disease (GBD) study. Accordingly, the economic burden of *Klebsiella pneumoniae* in Iran was assessed under four scenarios: 2021 GDP per capita (USD), 2021 GDP per capita × 3 (USD), 2021 GDP per capita (PPP), and 2021 GDP × 3 per capita (PPP). The percentage change in burden between two time points was calculated as:

$$\text{Percent}\;\text{Change}\;\left(\%\right)=\frac{{\text{Value}}_{2021}-{Value}_{2000}}{{\text{Value}}_{2000}}\times 100$$

To estimate the 95 % UI for the percentage change, we assumed that the values at each time point follow a normal distribution based on the reported mean and uncertainty bounds. We then performed a Monte Carlo simulation by drawing 1000 random samples from these distributions for each time point, calculating the percent change for each iteration, and using the mean and 2.5th and 97.5th percentiles of the simulated percent changes as the point estimate and 95 % UI, respectively.

In addition, we calculated the EAPC to quantify the temporal trend in burden over the study period. EAPC was derived by fitting a regression model to the natural logarithm of age-standardized rates over time:

$$\ln\left({Rate}_{t}\right)=\alpha +\beta \;.\;t$$

where *t* is the year, α is the intercept, and β is the slope of the regression line. The EAPC and its 95 % confidence interval were then calculated as:

$$EAPC=100\times \left(e^{\beta }-1\right)$$

A positive EAPC indicates an increasing trend, while a negative EAPC indicates a decreasing trend in the burden over time. This approach allowed us to compare the temporal trends of the burden in Iran with those in other regions.

All statistical analysis and visualizations were generated by Python (version 3.12.4). All figures in this study represent descriptive visualizations of the IHME-provided estimates and were not subjected to additional statistical testing for trend significance. Statistical significance of comparisons was determined based on the non-overlap of 95 % uncertainty intervals (UIs) or 95 % confidence intervals (CIs).

## 3 Results

### 3.1 Burden of *K*. *pneumoniae* AMR in Iran from 2000 to 2021

**Fig. 1:**
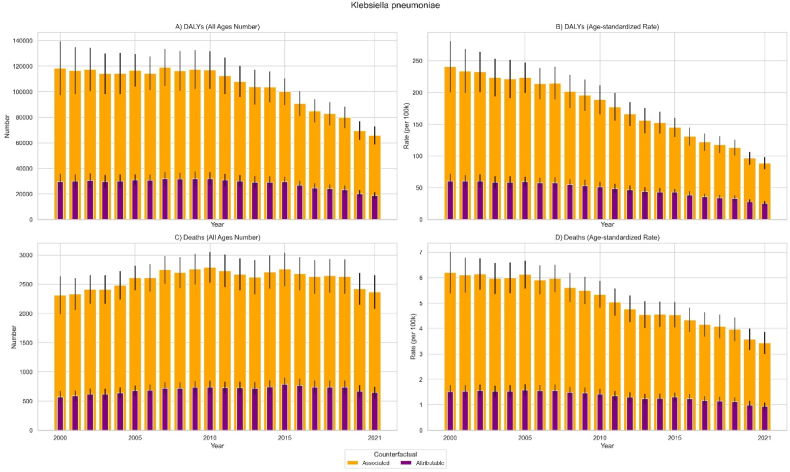
All-age numbers and age-standardized rates of Disability-Adjusted Life Years (DALYs) and deaths associated with and attributable to *Klebsiella pneumoniae* from 2000 to 2021.

In Iran, all-age deaths attributable to *K. pneumoniae* AMR increased from 565.9 (95 % UI 463.9 to 667.9) in 2000 to 643.5 (542.0–744.9) in 2021, representing a non-significant 14.7 % change (−10.4 to 45.4). Over the same period, all-age deaths associated with *K. pneumoniae* AMR moved from 2309.9 (1990.0–2629.8) to 2367.4 (2078.5–2656.3), representing a non-significant 3.0 % change (−14.7 to 23.6) (*Supplementary File*. Table 1 and Fig. 1).

In contrast, the Age-standardized rate (ASR) of deaths attributable to *K. pneumoniae* decreased by 39.6 % (−52.7 to −23.8), from 1.5 (1.3–1.8) in 2000 to 0.9 (0.8–1.1) per 100,000 people in 2021. The ASR of deaths associated with *K. pneumoniae* showed a decrease of 44.9 % (−54.4 to −34.0), falling from 6.2 (5.4–7.0) to 3.4 (3.0–3.9) per 100,000 people in the same period.

The burden of DALYs decreased. All-age DALYs attributable to *K. pneumoniae* AMR in Iran declined by 35.7 % (−50.0 to −17.2), from 29523.4 (23425.6–35621.2) in 2000–18787.1 (16176.7–21397.5) in 2021. All-age DALYs associated with *K. pneumoniae* AMR also declined by 44.0 % (−54.4 to −30.8), from 118506.1 (97460.2–139552.0) in 2000–65862.9 (58782.2–72943.6) in 2021.

The ASR of DALYs attributable to *K. pneumoniae* also decreased by 57.5 % (−66.8 to −45.7), falling from 60.2 (48.3–72.0) to 25.3 (21.7–28.9) per 100,000 people. Similarly, the ASR of DALYs associated with *K. pneumoniae* decreased by 62.8 % (−69.7 to −54.5), decreasing from 240.5 (200.2–280.9) to 88.7 (78.9–98.6) per 100,000 people.

When comparing the trend of *K. pneumoniae* AMR between Iran and other locations, the decrease in the ASR of associated mortality in Iran (EAPC: 2.61 %, 95 % CI: 2.77 % to −2.46 %) was similar to that in Saudi Arabia (−2.44 %, −2.53 % to −2.34 %), Türkiye (−3.24 %, −3.74 % to −2.73 %), and High-income countries (−2.32 %, −2.47 % to −2.16 %). Conversely, Iran's decline was significantly faster than the trends observed in Iraq (−2.03 %, −2.26 % to −1.79 %), Egypt (−2.11 %, −2.28 % to −1.94 %), the North Africa and Middle East region (−2.26 %, −2.38 % to −2.14 %), and the global average (−1.86 %, −1.92 % to −1.79 %) **(*Supplementary File.*** Table 2***)***.

### 3.2 Infectious syndromes

**Fig. 2:**
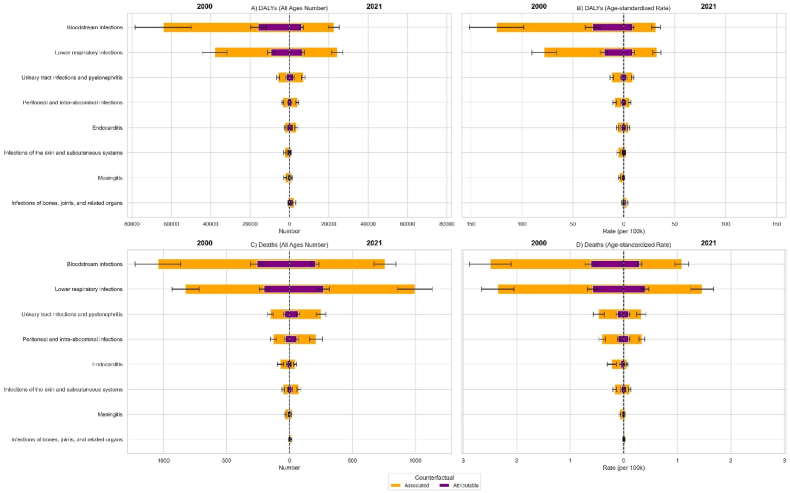
Disability-adjusted life years (DALYs) and deaths associated with and attributable to *K. pneumoniae* infectious syndromes in 2000 and 2021, presented as all-age numbers and age-standardized rates per 100,000 population.

In 2021, more than 70 % of deaths attributable to or associated with *K*. *pneumoniae* were caused by lower respiratory infections (LRIs) or bloodstream infections (BSIs) (Fig. 2). The burden associated with BSI decreased between 2000 and 2021, with the all-age number of deaths declining from 1043.7 (95 % UI: 862.3 to 1225.2) to 758.1 (671.9–844.3) and the ASR falling from 2.49 (2.10–2.88) to 1.09 (0.96–1.21) per 100,000. While the attributable ASR for BSI also decreased from 0.61 (0.49–0.73) to 0.30 (0.25–0.34) per 100,000, the change in the all-age number of attributable deaths was not significant, moving from 255.7 (203.2–308.2) to 206.1 (176.2–235.9).

The all-age number of deaths both associated with and attributable to LRI showed non-significant changes. Associated deaths moved from 825.0 (717.6–932.4) to 995.8 (858.1–1133.5), and attributable deaths from 202.1 (166.7–237.5) to 270.7 (223.7–317.6). Despite the stable absolute burden, the ASR for both associated deaths—2.35 (2.05–2.66) to 1.47 (1.25–1.68) per 100,000—and attributable deaths—0.58 (0.48–0.68) to 0.40 (0.33–0.47) per 100,000—decreased significantly.

Meningitis demonstrated a reduction across associated and attributable absolute deaths and ASRs. Infections of the skin and subcutaneous systems, urinary tract infections, and peritoneal and intra-abdominal infections all increased in the absolute number of both associated and attributable deaths, while their corresponding ASRs did not change significantly.

Trend of DALYs were more variable. For BSI and meningitis, both the absolute number and ASR of DALYs associated with and attributable to *K. pneumoniae* decreased. For LRI, absolute number of associated DALYs and both associated and attributable ASRs decreased; however, the change in the absolute number of attributable DALYs was not significant. The detailed changes of infectious syndromes are included in ***Supplementary File.*** Table 1**.**

### 3.3 Age pattern

**Fig. 3:**
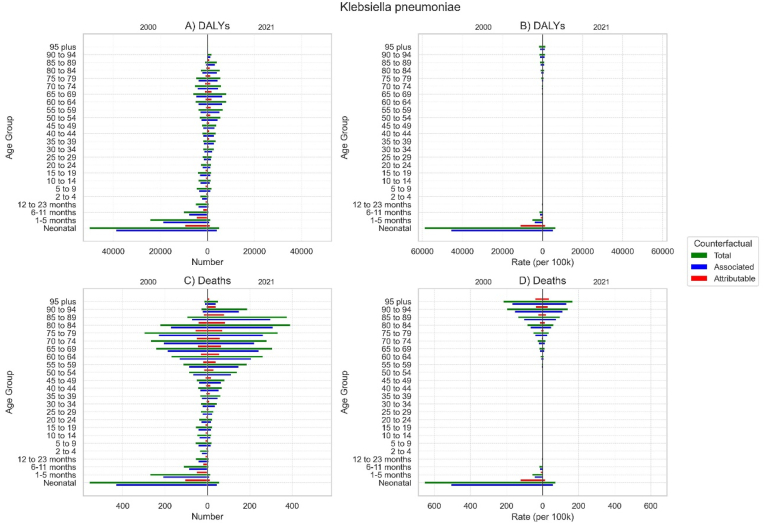
All-age numbers and age-standardized rates of disability-adjusted life years (DALYs) and deaths associated with and attributable to *Klebsiella pneumoniae* in 2000 and 2021, stratified by age group.

From 2000 to 2021, the absolute number of deaths and DALYs attributable to or associated with *K. pneumoniae* AMR in Iran decreased in individuals younger than 30 years and increased in those older than 30 years (Fig. 3). While age-specific rates decreased across most age-groups, these reductions were not significant for the 20-34-year range for death and the 20-39-year range for DALYs, in addition to those 85 years and older.

In 2000, neonates experienced the highest deaths, with a total of 558.4 (95 % UI 395.5 to 721.3). By 2021, this number decreased to 57.6 (39.0–76.2) deaths, and the age-specific mortality rate declined from 655.8 (464.5–847.1) to 73.3 (49.6–97.0) per 100,000 people. Conversely, the 80-to-84-year age group experienced the largest increase in the absolute number of deaths, rising from 223.5 (189.5–257.5) in 2000 to 392.1 (333.5–450.8) in 2021. This rise largely reflects a demographic shift, as the age-specific death rate for this group decreased from 86.2 (73.1–99.4) to 61.3 (52.1–70.4) per 100,000 people.

A similar pattern was observed for DALYs. The total DALYs for neonates decreased by approximately 10-fold, from 50265.2 (35604.0–64926.4) to 5192.6 (3514.5–6870.7) with a similar trend in ASR. In contrast, the absolute DALY burden for the 65-to-69-year age group increased from 6185.5 (5452.6–6918.4) to 8141.3 (7285.6–8997.0). However, its age-specific DALY rate decreased from 512.2 (451.5–572.9) to 345.4 (309.1–381.7) per 100,000 people.

### 3.4 Resistance patterns

**Fig. 4:**
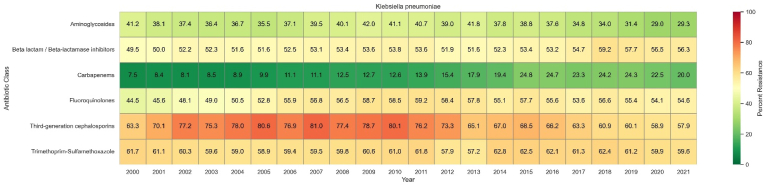
Trends in antibiotic resistance (%) for *Klebsiella pneumoniae* from 2000 to 2021 across different antibiotic classes. Colors represent the percentage of resistance, with green indicating lower resistance and red indicating higher resistance**.**

**Fig. 5:**
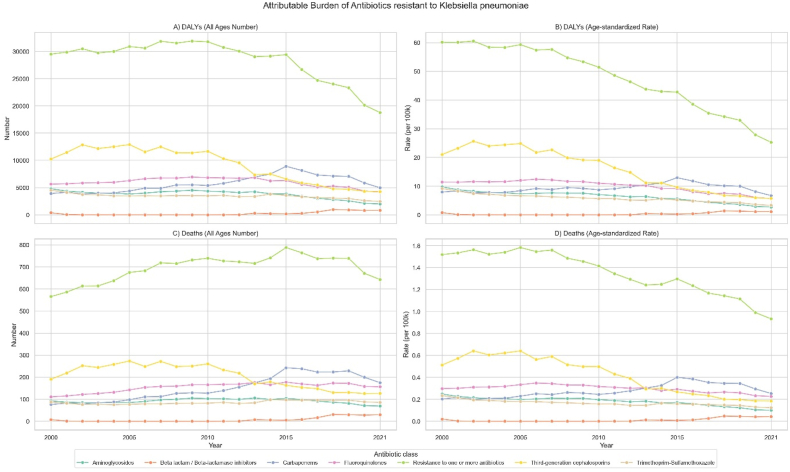
All-age numbers and age-standardized rates of disability-adjusted life years (DALYs) and deaths attributable to antibiotic-resistant *Klebsiella pneumoniae* from 2000 to 2021, stratified by antibiotic class.

In 2021, the highest resistance to *K*. *pneumoniae* in decreasing order belonged to: trimethoprim-sulfamethoxazole, third-generation cephalosporins, beta lactam/beta-lactamase inhibitors, fluoroquinolones, aminoglycosides, and carbapenems (Fig. 4). Carbapenems resistance increased from 7.5 % (95 % UI 6.2 to 8.9) in 2000 to 20.0 % (17.1–22.8) in 2021, resulting in the highest attributable all-age deaths (from 76.1 [54.4 to 97.9]) in 2000 to 175.0 [133.8 to 216.2] in 2021) among antimicrobial resistances (Fig. 5). In contrast, all-age deaths attributable to third-generation cephalosporins resistance decreased from 190.8 (127.0–254.7) in 2000 to 126.9 (82.6–171.2) in 2021.

### 3.5 Economic burden

From 2000 to 2021, economic burden attributable to *K. pneumoniae* declined from 93.6 to 280.8 [(95 % UI 74.3 to 112.9) to (222.8–338.8)] million USD to 84.4 to 253.2 [(72.7–96.1) to (218.0–288.4)] million USD. Also, economic burden associated with *K. pneumoniae* AMR declined from 375.7 to 1127.2 [(309.0–442.5) to (927.0–1327.4)] million USD to 295.9 to 887.6 [(264.1–327.7) to (792.19–983.03)] million USD. Considering PPP, in 2021, economic burden attributable to *K. pneumoniae* AMR was estimated at 291.1 to 873.31 [(250.7–331.6) to (751.97–994.65)] million USD PPP, and economic burden associated with *K. pneumoniae* AMR was estimated at 1020.5 to 3061.61 [(910.8–1130.3) to (2732.47–3390.75)] million USD PPP **(*Supplementary File.*** Fig. 6**)**.

## 4 Discussion

In this study, we discovered that from 2000 to 2021, the ASR of deaths and DALYs attributable to or associated with *K. pneumoniae* AMR significantly decreased in Iran, particularly among children. The growing burden on adults over 30 reflects a demographic shift. Although age-specific mortality and DALY rates decreased across most age groups, the decrease was not significant for those over 85 years old. BSIs were replaced by in 2021, and third-generation cephalosporin resistance was replaced by carbapenem resistance in 2021 as the leading causes of deaths and DALYs due to *K. pneumoniae* AMR in Iran. Despite a slight decrease in the economic burden of *K. pneumoniae* AMR over this period, the overall burden remains substantial.

The burden of *K. pneumoniae* AMR in Iran reflects global pattern of increasing impact on older adults and decreasing burden among children [5]. While trends in *K. pneumoniae* AMR-associated mortality in Iran were more favorable than the North Africa and Middle East regional and global averages, the country is still unlikely to meet the “10-20-30 targets by 2030.” [16]. This target, aiming for a 10 % reduction in all-age AMR-related deaths, is unlikely to be met, a challenge worsened by Iran's aging population.

Importantly, age-specific rates of death and DALYs in adults over 85 is stable in Iran, which requires careful interpretation as mortality in this population is frequently attributed to underlying comorbidities like cancer or heart failure, potentially masking the rising toll of resistant infections [17]. The elevated number of all-age deaths associated with *K. pneumoniae* LRI might also partly reflect these demographic changes, as AMR capabilities of the respiratory system microbiome intensify in older patients [18], and these patients are generally more affected by this condition. Alternatively, the trend may stem from attribution of secondary sepsis mortality to primary pneumonia, underreporting of milder lower respiratory infection cases, and more variable diagnostic criteria for these infections [19,20]. The increasing trend in UTIs, intraabdominal, skin, and joint *K. pneumoniae* infections, while partly attributable to demographic shifts, may impose a significant burden on the healthcare system through prolonged hospitalizations, more intensive resource utilization, and the use of last-resort antibiotics [[21], [22], [23]].

In contrast, the substantial reduction in *K. pneumoniae* AMR burden among children and young adults reflects improvements in water, sanitation, and hygiene (WASH) programs, as noted in UNICEF reports [24,25]. Previous research has demonstrated that WASH interventions significantly reduce infection rates, the need for antibiotics, and the horizontal transfer of resistance genes between species [16,[26], [27], [28]]. However, despite these improvements, neonatal mortality due to *K. pneumoniae* AMR still remains a significant contributor to DALYs and economic burden, which demands active neonatal intensive care unit surveillance and mandatory antimicrobial stewardship to guide empirical therapy and prevent outbreaks [29].

Deaths associated with third-generation cephalosporin *K. pneumoniae* resistance declined by approximately 50 % from 2000 to 2021, but unfortunately, it was replaced by carbapenem resistance, which increased by nearly 67 % over the past two decades. Our results showed a decline in deaths associated with resistant *K. pneumoniae* BSI, but the rising carbapenem resistance in *K. pneumoniae* might negatively impact this trend soon, as previous studies have shown the increased mortality in cases of carbapenem-resistant bloodstream infection [30,31].

The COVID-19 pandemic likely exacerbated the rise of carbapenem and multidrug-resistant (MDR) *K. pneumoniae* in Iran, undermining national efforts to preserve antibiotic effectiveness [32,33]. Studies during the pandemic noted a significant increase in *K. pneumoniae* isolates with high resistance rates (50 % to carbapenems; up to 96.3 % MDR [34]) [32,[34], [35], [36]]. This trend has been observed in other countries and was driven by widespread antibiotic use and strained infection control, particularly among COVID-19 patients with comorbidities and susceptibility to co-infections, which facilitated the spread of resistant strains and complicating treatment [32,33,[35], [36], [37], [38]].

The COVID-19 pandemic has also promoted the emergence of *K. pneumoniae* strains that are both hypervirulent and antimicrobial-resistant, which is a significant clinical hazard. While hypervirulent strains are typically less resistant, studies in Iran report their resistance rates are similar or even higher than classical strains [[39], [40], [41], [42]]. This convergence, which complicates patient management and raises mortality, demands surveillance that combines genomic tracking of virulence factors and antimicrobial resistance monitoring.

Addressing the divergent demographic, and changing *K. pneumoniae* antibiotic resistance pattern in Iran requires a systematic approach including enhanced neonatal care, alongside with rigorous infection prevention and control strategies for older adults at the hospitalization [43,44], rapid identification of resistant strains, development of effective empiric antibiotic protocols, and targeted treatment strategies [45]. The increasing resistance to carbapenems is specifically concerning in Iran, and it is not only limited to *K. pneumoniae* infections, as several studies call for immediate action [46,47]. This issue underscores the urgent need for national-level intervention, promoting rational carbapenems prescribing practices, stronger antibiotic stewardship programs, stricter regulatory guidelines, continuous surveillance to combat AMR in outpatient settings, and development or importation of novel, effective antibiotics. *Unfortunately, K. pneumoniae* AMR combat in Iran faces significant challenges due to widespread, unsupervised, and inappropriate outpatient antibiotic prescriptions, often for viral infections [48]. The overuse of “Watch” antibiotics—known to promote resistance [49] —and “Reserve” antibiotics, which should be reserved as last-line treatments, poses a major public health threat. Additionally, regional and international collaborations are essential for combating AMR and improving its effectiveness [50], especially as lower-to middle-income countries like Iran are more prone to be affected by AMR consequences [51].

Our study has several strengths that enhance its reliability. First, it utilizes data from the GBD 2021 study, one of the most comprehensive and up-to-date global health datasets, ensuring high-quality and consistent data for Iran. Second, it employs age-adjusted rates, which allow for accurate comparisons despite changes in population structure. Third, the study provides an economic assessment of *K. pneumoniae* AMR burden, demonstrating its direct impact on the national healthcare system.

However, this study has several limitations inherent to its reliance on modeled estimates. First, our findings are dependent on the primary data available for Iran within the GBD project's database. In instances of sparse local data, the model borrows strength from regional trends, which may not fully capture country-specific dynamics. Second, the input data are subject to potential surveillance biases. A reliance on hospital-based laboratory data may lead to an over-representation of severe, inpatient infections and an underestimation of the burden in community settings. Furthermore, potential inconsistencies in diagnostic and reporting practices across Iranian provinces over the study period could affect the accuracy of the estimated trends. Third, our national-level estimates mask significant sub-national heterogeneity. AMR burden and drivers can vary substantially across different provinces, limiting the direct application of these findings for local-level health planning, which requires granular surveillance. Additionally, our economic burden assessment uses GDP per capita as a proxy for the monetary value of a DALY. This approach does not account for Iran-specific healthcare costs, productivity losses, or variations in income distribution, and therefore provides an approximate rather than precise estimate of the true economic impact.

Future studies should evaluate the level of commitment to, and effectiveness of antibiotic stewardship programs in controlling *K. pneumoniae* AMR, especially resistance to carbapenems across different hospitals in Iran, as ASPs are cost-effective, and efficient ways of tackling microbial resistance [52]. Additionally, studied should focus on the genetic variations of *K. pneumoniae* AMR in Iran, and the role of horizontal gene transfer, evaluating their contributions to carbapenems resistance patterns. Estimating AMR trends in Iran could provide valuable insights for policymakers, enabling them to develop proactive strategies. Importantly, further investigations are needed to understand the rising burden of *K. pneumoniae* AMR among older adults and to identify underlying risk factors contributing to heightened resistance in this demographic.

## 5 Conclusion

The burden of *K. pneumoniae* AMR in Iran has grown, particularly among older adults. Despite advances in neonatal care, mortality and economic burden in this age group remain significant. BSI and third-generation cephalosporin resistance were replaced by and carbapenem resistance as the leading cause of burden due to *K. pneumoniae* AMR in Iran. Emergence of resistant, hypervirulent strains is a key concern in Iran. The implementation of WASH programs should continue with rigor, and policies must enforce antibiotic stewardship, particularly for carbapenems, which have shown a significant increase in resistance during the last two decades. Strict regulations on outpatient antibiotic prescriptions are also necessary. Additionally, there is an urgent need for the development of updated empiric treatment protocols and the introduction of improved antibiotics to combat AMR effectively.

## CRediT authorship contribution statement

**Amirhossein Shahsavand:** Writing – review & editing, Writing – original draft, Methodology, Funding acquisition, Formal analysis, Data curation. **Ali Golestani:** Writing – review & editing, Writing – original draft, Visualization, Validation, Supervision, Methodology, Investigation, Formal analysis, Data curation. **Samaneh Akbarpour:** Writing – review & editing, Validation, Software, Resources, Methodology, Funding acquisition. **Mohammadreza Salehi:** Writing – review & editing, Visualization, Validation, Supervision, Project administration, Methodology, Funding acquisition, Conceptualization. **Arash Seifi:** Writing – review & editing, Visualization, Validation, Methodology, Investigation. **Keyhan Mohammadi:** Writing – review & editing, Validation, Methodology, Investigation. **Maryam Shafaati:** Writing – review & editing, Visualization, Validation, Supervision, Project administration, Methodology, Funding acquisition, Conceptualization.

## Ethics approval

The findings are derived from estimates provided by the ARM 2021 study and comply with applicable guidelines and regulations. This study was approved by the research ethics committees of the Institute of Pharmaceutical Sciences at Tehran University of Medical Sciences (IR.TUMS.TIPS.REC.1403.115).

## Data availability statement

Not applicable.

## Funding

The Research Centre for Antibiotic Stewardship and Antimicrobial Resistance (RCASAR), Tehran University of Medical Sciences (TUMS), provided funding for the study under grant number 73294.

## Declaration of competing interest

The authors declare that they have no known competing financial interests or personal relationships that could have appeared to influence the work reported in this paper.
