# Supplementary material for: Burden of antimicrobial resistance and estimated economic impact of Klebsiella pneumoniae in Iran— A 2000 to 2021 analysis
Source: New Microbes New Infect. 2025 Nov 20;68:101675. doi: 10.1016/j.nmni.2025.101675 (PMC12686640; doi:10.1016/j.nmni.2025.101675)
Supplement: Multimedia component 1 [file mmc1.docx]

***Supplementary File V2.***

**Table 1.** Burden of *Klebsiella* *pneumoniae* antimicrobial resistance in Iran.

| **Measure** | **Metric** | **Associated** | | | **Attributable** | | |
| --- | --- | --- | --- | --- | --- | --- | --- |
|  |  | **2000** | **2021** | **Percent change (%)** | **2000** | **2021** | **Percent change (%)** |
| **DALYs** | **All ages number** | 118506.1 (97460.2 to 139552.0) | 65862.9 (58782.2 to 72943.6) | -44.0 (-54.4 to -30.8) | 29523.4 (23425.6 to 35621.2) | 18787.1 (16176.7 to 21397.5) | -35.7 (-50.0 to -17.2) |
| **DALYs** | **All ages rate** | 178.9 (147.1 to 210.6) | 77.2 (68.9 to 85.5) | -56.5 (-64.6 to -46.2) | 44.6 (35.4 to 53.8) | 22.0 (19.0 to 25.1) | -50.1 (-61.2 to -35.7) |
| **DALYs** | **Age-standardized rate** | 240.5 (200.2 to 280.9) | 88.7 (78.9 to 98.6) | -62.8 (-69.7 to -54.5) | 60.2 (48.3 to 72.0) | 25.3 (21.7 to 28.9) | -57.5 (-66.8 to -45.7) |
| **Deaths** | **All ages number** | 2309.9 (1990.0 to 2629.8) | 2367.4 (2078.5 to 2656.3) | 3.0 (-14.7 to 23.6) | 565.9 (463.9 to 667.9) | 643.5 (542.0 to 744.9) | 14.7 (-10.4 to 45.4) |
| **Deaths** | **All ages rate** | 3.5 (3.0 to 4.0) | 2.8 (2.4 to 3.1) | -19.6 (-33.8 to -2.8) | 0.9 (0.7 to 1.0) | 0.8 (0.6 to 0.9) | -10.4 (-31.3 to 14.1) |
| **Deaths** | **Age-standardized rate** | 6.2 (5.4 to 7.0) | 3.4 (3.0 to 3.9) | -44.9 (-54.4 to -34.0) | 1.5 (1.3 to 1.8) | 0.9 (0.8 to 1.1) | -39.6 (-52.7 to -23.8) |

**Table 2.** Trend of age-standardized death rate associated with *Klebsiella* *pneumoniae* antimicrobial resistance in Iran and selected countries and regions.

| **Location** | **2000** | **2021** | **EAPC (%)** |
| --- | --- | --- | --- |
| **Iran** | 6.2 (5.4 to 7.0) | 3.4 (3.0 to 3.9) | -2.61 (-2.77 to -2.46) |
| **Saudi Arabia** | 10.0 (8.4 to 11.5) | 5.9 (4.8 to 6.9) | -2.44 (-2.53 to -2.34) |
| **Türkiye** | 7.7 (6.6 to 8.8) | 4.7 (3.9 to 5.5) | -3.24 (-3.74 to -2.73) |
| **Iraq** | 9.3 (7.6 to 11.1) | 5.9 (4.8 to 7.1) | -2.03 (-2.26 to -1.79) |
| **Egypt** | 17.2 (15.5 to 18.9) | 11.4 (9.5 to 13.3) | -2.11 (-2.28 to -1.94) |
| **North Africa and Middle East** | 10.8 (9.4 to 12.1) | 6.8 (5.9 to 7.7) | -2.26 (-2.38 to -2.14) |
| **High-income** | 2.5 (2.1 to 2.8) | 1.6 (1.4 to 1.8) | -2.32 (-2.47 to -2.16) |
| **Global** | 11.6 (10.4 to 12.9) | 7.4 (6.6 to 8.2) | -1.86 (-1.92 to -1.79) |


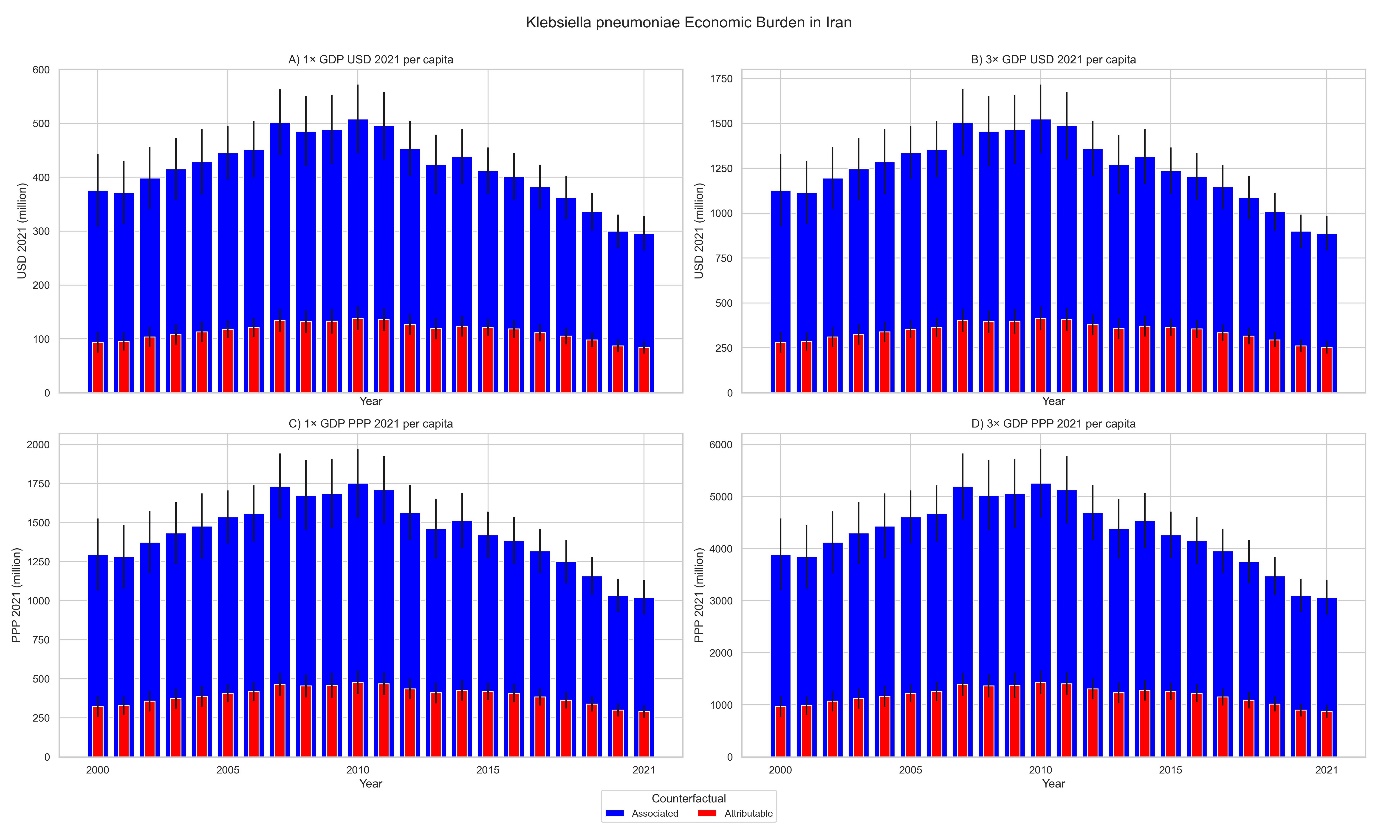


**Figure 6.** Economic burden of *Klebsiella* *pneumoniae* in Iran from 2000 to 2021, estimated using GDP per capita in united states dollar (USD) and purchasing power parity (PPP). Costs were calculated at 1× and 3× GDP per capita in both USD2021 and PPP. Error bars indicate uncertainty intervals.

**Data processing and modelling**

**Sepsis and infectious syndromes (steps 1 and 2)**

To estimate the number of sepsis-related deaths, cause-of-death data from the GBD 2021 study were used to identify deaths by age, sex, and location in which sepsis appeared as part of the pathway to death. Sepsis was defined as a life-threatening organ dysfunction resulting from a dysregulated host response to infection. In the first modeling step, multiple sources of cause-of-death information were incorporated, including data from millions of death certificates, hospital discharge records, and mortality surveillance systems across several countries. Mixed-effects logistic regression models were then applied to estimate the proportion of sepsis occurring within each underlying cause of death. The predicted fractions of sepsis were multiplied by cause-, age-, sex-, year-, and location-specific mortality estimates from GBD to generate the mortality envelope used in this analysis. This mortality envelope included all sepsis deaths with non-infectious underlying causes as well as explicit sepsis deaths with infectious causes. Although deaths related to COVID-19 were included in the sepsis mortality envelope, they were not used in estimating the burden of antimicrobial resistance.

In modelling step two, details on the pathways of disease provided in the multiple cause of death data or hospital discharge data were used in a second stage of mixed effects logistic regression models to further subdivide sepsis deaths into 22 major infectious syndromes, of which 11 had both bacterial aetiologies for which we estimate AMR burden and sufficient data available for pathogen modelling (appendix 1 p 21). These regressions predicted the fraction of sepsis-related deaths that were caused by a given infectious syndrome, separately for each GBD underlying cause of death, location, age, sex, and year. We used this fraction to subdivide sepsis deaths in non-infectious underlying causes into specific infectious syndromes. For underlying causes of death that are themselves infectious, all deaths were assigned to a single corresponding infectious syndrome (eg, GBD cause “lower respiratory infections” was assigned to infectious syndrome “lower respiratory infections”; appendix 1 pp 22–24).

**Case fatality rates and pathogen distribution for deaths and incident cases (steps 3 and 4)**

In the third analytical step, data linking pathogen-specific disease incidence to deaths were used to develop models for pathogen-specific case-fatality ratios (CFRs) that varied by age, year, location, and infectious syndrome. Depending on the availability and quality of data, four levels of granularity were applied for each pathogen-syndrome: a unique model for that specific pathogen-syndrome, a unique intercept within a model covering all pathogens, a pooled model for each pathogen type (bacteria, fungus, parasite, or virus), or a fully pooled model using all data. CFRs were estimated using a regression model framework, incorporating the Healthcare Access and Quality Index and other covariates to adjust for potential biases. These CFR estimates were then used to infer the number of cases for each pathogen from mortality-only data sources, which still provided information on pathogen distributions. Overall, data from 24 million isolates and cases across 142 countries and territories were used to estimate the pathogen composition of each infectious syndrome by age, year, and location. To manage the diverse datasets, a multinomial estimation framework was applied that accommodates partial and composite observations, allows covariates in network analysis, and incorporates Bayesian priors. Where applicable, incidence and death proportions were estimated for viral, fungal, parasitic, and bacterial pathogens. However, the antimicrobial resistance (AMR) burden was calculated only for selected bacteria with clinically relevant resistance and sufficient data.

**Prevalence of resistance by pathogen (steps 5 to 7)**

The 84 pathogen–antibiotic class combinations were selected by first compiling a complete list of clinically relevant combinations with available data, then removing those that lacked sufficient data or were not feasible for accurate statistical modelling. Microbial datasets from collaborators were supplemented with aggregate data from systematic reviews and published surveillance reports. Resistance was classified into two categories: susceptible and non-susceptible, with the latter including dose-dependent, intermediate, and resistant interpretations. Combinations where bacteria have intrinsic resistance were excluded. About two-thirds of the datasets reported laboratory susceptibility interpretations rather than quantitative test values, and these interpretations were based on various guidelines, potentially introducing bias. For the remaining third, minimum inhibitory concentration (MIC) data were available and used to classify isolates according to the 2023 Clinical and Laboratory Standards Institute (CLSI) breakpoints, which served as the reference standard. MIC data also helped infer relationships between resistance proportions when different versions of CLSI or European Committee on Antimicrobial Susceptibility Testing guidelines were used, allowing for adjustment of data reported only as laboratory interpretations. Finally, to address bias from tertiary care facilities, resistance rates from these sites were adjusted by aligning them with data from non-tertiary and mixed healthcare settings.

After adjustments, a two-stage spatiotemporal modelling approach was used to estimate resistance prevalence for each pathogen–drug combination by location and year from 1990 to 2021. In the first stage, a stacked ensemble model was fitted using the input data and selected health-related covariates. In the second stage, the ensemble model estimates were smoothed over space and time using a spatiotemporal Gaussian process regression. Tuberculosis, including multidrug-resistant and extensively drug-resistant forms, was treated as an exception, relying on existing estimates.

Because antibiotic consumption strongly influences resistance, national-level antibiotic use was modelled and included as a covariate in the ensemble model. Data from Demographic and Health Surveys, Multiple Indicator Cluster Surveys, and pharmaceutical sales from IQVIA, WHO, and the European Centre for Disease Prevention and Control were combined using an ensemble spatiotemporal Gaussian process regression. This produced location-year estimates of antibiotic consumption for all 204 countries and territories from 1990 to 2021.

To address multidrug resistance, line-level microbiology data testing the same isolates against multiple antibiotics were used to calculate both marginal resistance frequencies and pairwise co-occurrences. Using this information, a multinomial distribution that best matched the observed data in a least squares sense was derived. This method ensures a solution even if the data are noisy or inconsistent, providing an approximate distribution that aligns with the observed margins. This approach was applied for each location-year combination for all antibiotics analyzed.

**Relative risk of death for drug-resistant infection compared with drug-sensitive infections (steps 8 and 9)**

Using data from 1,238 sources covering 296 million patient samples with outcomes and resistance information, we estimated the relative risk of death for each pathogen–drug combination in resistant versus drug-sensitive infections. Due to limited data, these estimates were calculated by antibiotic class, pathogen, and infectious syndrome, assuming that risk did not vary by location or age. A two-stage modelling approach was applied, combining mixed-effects binomial logistic regression and mixed-effects meta-regression to determine the relative risk of death for each combination. For non-fatal outcomes, we estimated the additional length of hospital stay associated with resistant infections compared to susceptible infections of the same type. Data for this came from 309 sources covering 38 million admissions. A similar two-stage nested mixed-effects meta-regression framework was used to model excess length of stay. For drug-resistant *Neisseria gonorrhoeae*, only non-fatal estimates were produced due to limited data. To calculate the overall burden of multiple pathogen–drug combinations that were mutually exclusive within a given pathogen, population attributable fractions (PAFs) were generated for each resistance profile involving resistance to at least one drug.

**Computing burden attributable to drug resistance and burden associated with drug-resistant infections (step 10)**

We calculated two counterfactual scenarios to estimate the burden of drug-resistant infections: one representing the burden attributable to bacterial AMR compared with drug-sensitive infections, and another representing the burden associated with AMR compared with no infection. To estimate the burden attributable to AMR, we first calculated deaths due to resistance by multiplying, for each underlying cause, the cause-specific deaths by the fraction occurring with sepsis, the fraction of sepsis deaths attributable to each infectious syndrome, the fraction of syndrome deaths attributable to each pathogen, and the mortality population attributable fraction (PAF) for each resistance profile. Age-specific deaths were converted into years of life lost (YLLs) using standard life expectancy. For years lived with disability (YLDs), we multiplied infectious syndrome incidence by the fraction of cases attributable to each pathogen, the YLD per case, and the non-fatal PAF. For resistance profiles affecting multiple antibiotic classes, burden was proportionally distributed across the relevant classes based on excess risk, ensuring mutually exclusive estimates for each pathogen–drug combination. Disability-adjusted life years (DALYs) were calculated by summing YLLs and YLDs. The overall AMR burden under the drug-sensitive counterfactual was obtained by summing the burden across all pathogen–drug combinations. To estimate the fatal burden of AMR, the same method was applied, replacing the mortality PAF with the prevalence of resistance in deaths.
